# Diaphragm Dysfunction and ICU-Acquired Weakness in Septic Shock Patients with or without Mechanical Ventilation: A Pilot Prospective Observational Study

**DOI:** 10.3390/jcm12165191

**Published:** 2023-08-09

**Authors:** Yuta Takahashi, Tomoyuki Morisawa, Hiroshi Okamoto, Nobuto Nakanishi, Noriko Matsumoto, Masakazu Saitoh, Tetsuya Takahashi, Toshiyuki Fujiwara

**Affiliations:** 1 Department of Rehabilitation Medicine, Juntendo University Graduate School of Medicine, Tokyo 113-8421, Japan; yuta@luke.ac.jp (Y.T.); t-fujiwara@juntendo.ac.jp (T.F.); 2 Department of Rehabilitation, St. Luke’s International Hospital, Tokyo 104-8560, Japan; 3 Department of Physical Therapy, Faculty of Health Science, Juntendo University, Tokyo 113-8421, Japan; m.saito.tl@juntendo.ac.jp (M.S.); te-takahashi@juntendo.ac.jp (T.T.); 4 Department of Critical Care Medicine, St. Luke’s International Hospital, Tokyo 104-8560, Japan; hiroshi.okamota.1121@gmail.com; 5 Division of Disaster and Emergency Medicine, Department of Surgery Related, Kobe University Graduate School of Medicine, Kobe 650-0017, Japan; nobuto_nakanishi@yahoo.co.jp; 6 Department of Nutrition, St. Luke’s International Hospital, Tokyo 104-8560, Japan; matsuri@luke.ac.jp

**Keywords:** diaphragm, intensive care, sepsis: septic shock, mechanical ventilation

## Abstract

Sepsis is a risk factor for diaphragm dysfunction and ICU-acquired weakness (ICU-AW); however, the impact of mechanical ventilation (MV) on these relationships has not been thoroughly investigated. This study aimed to compare the incidence of diaphragm dysfunction and ICU-AW in patients with septic shock, with and without MV. We conducted a single-center prospective observational study that included consecutive patients diagnosed with septic shock admitted to the ICU between March 2021 and February 2022. Ultrasound measurements of diaphragm thickness and manual measurements of limb muscle strength were repeated after ICU admission. The incidences of diaphragm dysfunction and ICU-AW, as well as their associations with clinical outcomes, were compared between patients with MV and without MV (non-MV). Twenty-four patients (11 in the MV group and 13 in the non-MV group) were analyzed. At the final measurements in the MV group, eight patients (72.7%) had diaphragm dysfunction, and six patients (54.5%) had ICU-AW. In the non-MV group, 10 patients (76.9%) had diaphragm dysfunction, and three (23.1%) had ICU-AW. No association was found between diaphragm dysfunction and clinical outcomes. Patients with ICU-AW in the MV group had longer ICU and hospital stays. Among patients with septic shock, the incidence of diaphragm dysfunction was higher than that of ICU-AW, irrespective of the use of MV. Further studies are warranted to examine the association between diaphragm dysfunction and clinical outcomes.

## 1. Introduction

In recent years, the incidence of sepsis has been growing, with the number of sepsis survivors synergistically increasing [1,2]. In addition, an increasing number of studies on the long-term prognosis of sepsis survivors have emerged, suggesting that sepsis is not just a transient acute illness but also a disease with long-term health consequences [3]. Sepsis is a significant risk factor for developing profound muscle weakness called ICU-acquired weakness (ICU-AW), a prognostic indicator for critically ill ICU patients [4]. On the other hand, it has recently been recognized that sepsis causes functional impairment not only to the peripheral muscles but also to the diaphragm, which is the main respiratory muscle. Furthermore, respiratory sarcopenia, defined as the age-related deterioration of respiratory muscle function [5], may be observed more frequently in aged patients with sepsis. Regarding these respiratory muscle assessment modalities, noninvasive assessment using ultrasound is becoming feasible and being performed as part of point-of-care ultrasound in the ICU [6].

Previous studies have reported that diaphragm atrophy occurs in around 40–50% of critically ill patients [7,8], and diaphragm dysfunction occurs in about 50–60% [9,10]. Moreover, patients with sepsis are more susceptible to progressive diaphragm atrophy and dysfunction than non-septic patients in the ICU [11,12,13]. As diaphragm atrophy and dysfunction are associated with prognosis in septic mechanically ventilated patients [13,14,15], it is crucial to screen for those conditions in patients with sepsis to prevent morbidity. However, the majority of previous studies on diaphragm dysfunction have been conducted only in mechanically ventilated patients. Clinical data on diaphragm atrophy and dysfunction in non-mechanically ventilated patients with sepsis are limited. Investigating this knowledge gap could lead to a better understanding of the impact of mechanical ventilation (MV) on sepsis and diaphragm dysfunction, and identify patient groups to be considered for therapeutic intervention. Furthermore, previous studies have shown that diaphragm dysfunction develops in patients with ICU-AW [16,17]. Although diaphragm dysfunction in patients with sepsis may be associated with ICU-AW, there is a lack of research examining the association between diaphragm dysfunction and ICU-AW in patients with sepsis, with or without mechanical ventilation.

Therefore, this study aimed to compare diaphragm dysfunction in patients with septic shock with and without mechanical ventilation. Additionally, we investigated the overlap between diaphragm dysfunction and skeletal muscle dysfunction, and examined the association between diaphragm dysfunction and clinical outcomes.

## 2. Materials and Methods

### 2.1. Study Design and Participants

This single-center prospective observational study was conducted at St. Luke’s International Hospital, a 520-bed teaching hospital in Tokyo, between March 2021 and February 2022.

We included all consecutive patients diagnosed with septic shock admitted to the ICU for ≥48 h and were available for follow-up diaphragm ultrasound measurements. Patients with septic shock were identified based on the following criteria described in the Third International Consensus Definitions for Sepsis and Septic Shock (Sepsis-3) [18], which requires a clinical presentation of sepsis with persisting hypotension requiring vasopressors to maintain a mean arterial pressure of at least 65 mm Hg and a serum lactate level of more than 2 mmol/L (18 mg/dL), despite adequate volume resuscitation. Patients under 18 years of age, those with a history of neuromuscular or diaphragm disorders, and those who spent more than a week in the general ward prior to ICU admission were excluded from the study. Patients who were mechanically ventilated for >72 h were classified into the MV group, whereas those who were not ventilated were classified into the non-MV group.

This study was conducted in accordance with the Strengthening the Reporting of Observational Studies in Epidemiology (STROBE) guidelines, and approved by the Ethics Committee of St. Luke’s International Hospital (date of approval: 12 November 2020, approval number: 20-R155). The Ethics Committee waived the need for informed consent. All the procedures were performed in accordance with the principles of the Declaration of Helsinki.

### 2.2. Measurements of Diaphragm Thickness

We used a portable ultrasound device (iViz air ver.4, Fujifilm, Tokyo, Japan) with a 5–10 MHz linear transducer. Imaging was performed by B-mode ultrasonography. As previously reported [19], measurements of diaphragm thickness were performed in the zone of apposition between the eighth and tenth right intercostal spaces and between the antero-axillary and midaxillary lines. Expiratory diaphragm thickness was measured at the end of the expiratory phase, and inspiratory diaphragm thickness was measured at the end of the inspiratory phase. The average of three respiratory cycles was used for the analysis. The thickening fraction (TF) of the diaphragm was calculated as [thickness at inspiration − thickness at expiration]/[thickness at expiration] × 100, as in previous research [20,21]. One measurement of diaphragm thickness was repeated on ICU days <3, 3–5, 5–7, 7–10, and 10–14 by the same staff (YT) experienced in diaphragm ultrasound.

### 2.3. Data Definition and Collection

The primary outcome was the prevalence of diaphragm dysfunction. Diaphragm dysfunction was defined as a TF of less than 29%, based on a previous study [22]. Secondary outcomes were changes in diaphragm thickness and TF over time. The Medical Research Council (MRC) score was assessed repeatedly at the same time as the diaphragm ultrasound when the patient was awake and able to follow the instructions by the same staff. ICU-AW was diagnosed with an MRC sum score of less than 48 when all muscle groups were measurable; the average MRC score of the evaluable limb was less than 4 if there is a limb that cannot be evaluated owing to trauma or pain; symmetrical alteration in muscle strength mainly in the proximal regions of the limbs and causes of muscle weakness not associated with critical illness were excluded [23]. Weaning failure was defined as the need for reintubation within 48 h following extubation. Patient data, including clinical history, laboratory results, vital signs, clinical treatment, and rehabilitation outcomes, were collected from the electronic medical records of our institution.

### 2.4. Statistical Analysis

Continuous variables are expressed as median (interquartile range) for data with normal and non-normal distributions, respectively, while categorical variables are described as numbers (percentages). The Mann–Whitney U-test and χ^2^-test were used to compare continuous and categorical variables between the two groups. Due to the exploratory nature of the research, the sample size was not calculated a priori but was determined based on a feasible size. All reported *p* values were two-sided, and statistical significance was set at *p* values < 0.05. All analyses were performed using SPSS for Microsoft Windows (version 24.0; SPSS, Armonk, NY, USA).

## 3. Results

Of the 24 patients, 11 were mechanically ventilated and classified as the MV group, while the remaining 13 were classified as the non-MV group. The number of patients on MV in the MV group, the number of patients using the assist-control mode, the number of patients using pressure-support mode, and the median inspiratory support pressure were, respectively, *n* = 11, *n* = 9, *n* = 1, and 11 cm H_2_O on ICU day < 3; *n* = 7, *n* = 5, *n* = 2, and 10 cm H_2_O on ICU days 3–5; *n* = 3, *n* = 1, *n* = 2, and 12 cm H_2_O on ICU days 5–7; *n* = 2, *n* = 0, *n* = 2, and 8.5 cm H_2_O on ICU days 7–10; and *n* = 1, *n* = 1, *n* = 0, and 13 cm H_2_O on ICU days 10–14. Patients in the MV group were of older age (*p* = 0.035), had a longer length of ICU stay (*p* = 0.005), and had a higher rate of systemic steroid usage (*p* = 0.015), as well as a higher rate of propofol (*p* < 0.001) and midazolam (*p* < 0.001) use compared to those in the non-MV group. Additionally, patients in the MV group had higher APACHE Ⅱ score (*p* = 0.004), SOFA score at ICU admission (*p* = 0.009), and maximum SOFA score in ICU (*p* < 0.001), as well as a higher incidence lung infection (*p* = 0.017) compared to the non-MV group (Table 1).

**Figure 1:**
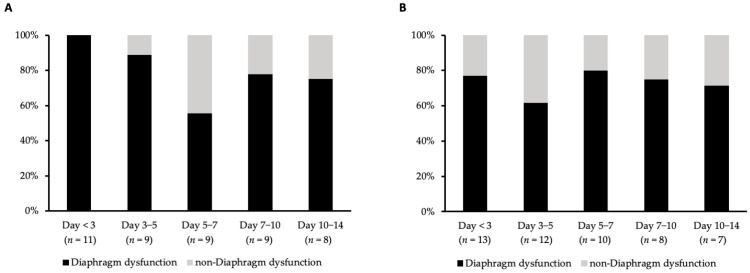
(**A**) Incidence of diaphragm dysfunction at each time point in the mechanical ventilation group. (**B**) Incidence of diaphragm dysfunction at each time point in the non-mechanical ventilation group.

A total of 97 ultrasound measurements were performed for all 24 patients. Although the number of cases that could be followed up decreased during the observation period, owing to patient discharges and missing measurements, all patients received at least three measurements. Among the MV group, the incidence of diaphragm dysfunction during the study period ranged from 56% to 100%, while the incidence ranged from 62% to 80% in the non-MV group during the follow-up period (Figure 1). The baseline measurements were obtained on a median day of 1.0 (with an interquartile range (IQR) of 1.0 to 1.3), while the final measurements were taken on a median day of 12.0 (IQR, 9.8 to 13.0) from ICU admission date. Final measurement dates were not significantly different between the MV and non-MV groups.

Diaphragm thickness at expiration in the MV group significantly decreased over time (*p* = 0.044), whereas no longitudinal change was observed in the non-MV group. The diaphragm thickening fraction in the MV group showed a significant increase over time (*p* = 0.042); however, the median value remained at 0%. No longitudinal changes were observed in the non-MV group (Table 2).

At the final measurements in the MV group, eight patients (72.7%) had diaphragm dysfunction, six patients (54.5%) had ICU-AW, and all patients who developed ICU-AW coexisted with diaphragm dysfunction. Moreover, at the final measurements in the non-MV group, ten patients (76.9%) had diaphragm dysfunction, three patients (23.1%) had ICU-AW, and all patients who developed ICU-AW coexisted with diaphragm dysfunction, as in the MV group (Table 3).

There were no significant differences in in-hospital mortality, number of patients transferred to rehabilitation hospitals, and length of ICU and hospital stay between patients with and without diaphragm dysfunction in the MV and non-MV groups (Table 4). In contrast, the number of patients transferred to rehabilitation hospitals and the lengths of ICU and hospital stay were significantly higher in the ICU-AW group than the group without ICU-AW among the MV group (*p* = 0.018, 0.004, and 0.004, respectively) (Table 4).

## 4. Discussion

This study aimed to compare diaphragm dysfunction in patients with septic shock with and without MV, leading to a better understanding of the impact of MV on sepsis and diaphragm dysfunction. At the final measurements (median, 12 days after ICU admission), diaphragm dysfunction was present in 72.7% of the MV group and 76.9% of the non-MV group, revealing a high rate of diaphragm dysfunction even in patients without MV. Furthermore, diaphragm dysfunction and ICU-AW overlapped each other, with the MV group having more diaphragm dysfunction than ICU-AW, while the non-MV group had three times more diaphragm dysfunction than ICU-AW. Meanwhile, the association between diaphragm dysfunction and clinical outcomes was poor, and the clinical impact of diaphragm dysfunction in patients with sepsis was unclear.

Our findings of a high incidence of diaphragm dysfunction in the non-MV group are in contrast to a previous study of sepsis patients, where 71% of the MV group and 37% of the non-MV group had diaphragm dysfunction at ICU admission [9]. One possible reason for the higher incidence of diaphragm dysfunction in our non-MV group is the greater proportion of intra-abdominal infections in these patients, which have been linked to a higher risk of diaphragm dysfunction in previous studies [9]. Additionally, our study exclusively included septic shock patients, which is a more severe type of sepsis and may explain the higher incidence of diaphragm dysfunction in the non-MV group. The decrease in diaphragm thickness in the MV group was consistent with previous studies [13,14,15]. We also observed a similar clinical course of recovery in the thickening fraction once it was reduced at the start of MV, as in previous studies [7]. There are two representative studies on the overlap between diaphragm dysfunction and ICU-AW [16,17]. These studies indicate that patients with ICU-AW have a high rate of diaphragm dysfunction (62–80%); however, patients with diaphragm dysfunction have a low rate of ICU-AW coexistence [16], meaning that diaphragm dysfunction often occurs alone. However, these previous studies have mainly focused on mechanically ventilated patients, likely to exhibit diaphragm-specific weakness. In contrast, our study was conducted in patients with sepsis, which can affect both limb muscles and the diaphragm, leading to a high incidence of overlap between diaphragm dysfunction and limb muscle weakness. In previous studies, the overlap was high among patients with septic shock [17], while there was limited overlap among fewer patients with shock [16]. Prior studies in mechanically ventilated patients have reported that diaphragm dysfunction is associated with poor prognosis [10,16]. However, our study found no association between diaphragm dysfunction and prognosis, consistent with a previous study of patients with sepsis [9]. Due to the limited sample size and insufficient number of outcomes in our study, it is not possible to conclude the association between diaphragm dysfunction and prognosis. Future large-scale studies are warranted to investigate the clinical impact of diaphragm dysfunction in non-MV patients with sepsis.

As described in previous studies, the mechanisms underlying diaphragm dysfunction include nutrition, metabolic disorders, sepsis, shock, surgery, immobilization, and mechanical ventilation [24]. Sepsis is thought to cause diaphragm dysfunction by impairing various levels of the energy supply chain, including changes in blood flow distribution and utilization of blood flow, and direct impairment of contractile proteins by the action of septic mediators as cytokines [25]. These mechanisms could explain the high rate of diaphragm dysfunction observed in the non-MV group in our study. In addition, patients resuscitated during the rescue phase of the septic shock may develop pulmonary congestion and respiratory effort due to volume overload [26]. In patients with respiratory effort, the intercostal and cervical muscles, which are respiratory accessory muscles, may be recruited for inspiratory effort, and the diaphragm may be relatively weakened. Sepsis is known to harm both respiratory and limb skeletal muscles, and sepsis-induced diaphragm dysfunction and ICU-AW are caused by similar pathways to some extent [16,17]. Therefore, the concurrent occurrence of the two muscle failures is easily predictable, but the cause of the higher rate of diaphragm dysfunction compared to ICU-AW is poorly understood. Several previous studies have reported a higher susceptibility of the diaphragm to the effects of bacteremia relative to the limb muscles [27,28]. Additionally, the local effects of intra-abdominal infection on the diaphragm have also been noted in previous studies [29], and the location of the primary infection site near the diaphragm may explain the greater number of dysfunctions in the diaphragm than in the limb muscles.

Diaphragm function assessment is still uncommon in clinical practice for ICU patients, and standard assessment methods, as well as clear cut-off values, still need to be determined. We demonstrated that diaphragm dysfunction could also occur in non-mechanically ventilated patients with septic shock, and it deserves the clinician’s attention, even if its role in patient outcomes is yet to be determined. Although managing diaphragm dysfunction has not yet been established, early passive standing [30], whole-body exercise [31], and inspiratory muscle training are expected to improve diaphragm function. This study provides basic data for future medical staff to assess diaphragm function and consider intervention strategies.

Although our study provides important insights into diaphragm dysfunction in patients with septic shock, several limitations must be considered. Firstly, this was a single-center study, which may limit the generalizability of our findings. Additionally, the small sample size may have limited clinical outcome investigation, which could have influenced the overall prognosis. Despite these limitations, our study contributes to the literature by following up with both mechanically ventilated and non-ventilated patients with septic shock, providing a more comprehensive understanding of diaphragm dysfunction in this population.

## 5. Conclusions

Sepsis is a major risk factor for diaphragm dysfunction, and our study showed that diaphragm dysfunction occurs at a high rate, even in patients without MV. Further analysis is warranted to investigate the clinical impact of diaphragm dysfunction in patients with sepsis without MV. Our study provides important insights into the need to evaluate diaphragm function in critically ill patients and consider early intervention strategies.

## Figures and Tables

**Table 1:** Clinical characteristics of the study population.

Variables		MV Group (*n* = 11)	non-MV Group (*n* = 13)	*p*-Value
Age, years		69.0 (66.0, 74.5)	80.0 (73.0, 87.0)	0.035
Male		6 (54.5)	8 (61.5)	1.000
BMI, kg/m^2^		24.4 (20.4, 28.3)	21.4 (18.5, 24.0)	0.119
Length of hospital stay, days		26.0 (15.0, 79.0)	21.0 (14.0, 37.0)	0.494
Length of ICU stay, days		10.0 (6.0, 22.0)	5.0 (4.0, 6.0)	0.005
Length of MV, days		5.0 (4.5, 16.0)		
Time to start MV, days		0 (0, 0)		
Surgical patients		2 (18.2)	0 (0)	0.199
Noradrenalin use		11 (100)	13 (100)	1.000
Vasopressin use		11 (100)	4 (30.8)	<0.001
Systemic steroid use		8 (72.7)	3 (23.1)	0.015
Propofol use		7 (63.6)	0 (0)	<0.001
Midazolam use		10 (90.9)	0 (0)	<0.001
Renal replacement therapy		3 (27.3)	0 (0)	0.082
Delirium incidence		8 (72.7)	7 (53.8)	0.423
Rehabilitation days, days		22.0 (11.5, 86.5)	15.0 (10.0, 30.0)	0.331
Time to start rehabilitation, days		1.0 (1.0, 1.0)	1.0 (1.0, 2.0)	0.392
Time to initial sitting, days		2.0 (2.0, 3.0)	4.0 (2.3, 6.5)	0.036
Time to initial ambulation, days		4.0 (3.0, 5.0)	6.5 (4.0, 13.3)	0.161
APACHE Ⅱ at ICU admission, points		26.0 (17.0, 30.0)	14.0 (12.0, 17.0)	0.004
SOFA score at ICU admission, points		11.0 (9.5, 13.0)	7.0 (6.0, 9.0)	0.009
Maximum SOFA score in ICU, points		13.0 (11.0, 14.5)	8.0 (8.0, 9.0)	<0.001
Clinical frail scale, points		4.0 (3.0, 5.0)	4.0 (4.0, 6.0)	0.228
Charlson comorbidity index, points		1.0 (0.5, 3.5)	2.0 (1.0, 4.0)	0.361
Site of infection	Lungs	5 (45.5)	0 (0)	0.017
	Abdomen	3 (27.3)	9 (69.2)	
	Urinary tract	3 (27.3)	3 (23.1)	
	Soft tissue and bone	0 (0)	1 (7.7)	

Values are expressed as median (interquartile range) or *n* (%). BMI, Body Mass Index; ICU, Intensive Care Unit; MV, mechanical ventilation; APACHE II, Acute Physiology and Chronic Health Evaluation II; SOFA, Sequential Organ Failure Assessment.

**Table 2:** Longitudinal changes in diaphragm thickness at expiration and thickening fraction from baseline to the final measurement.

	Thickness at Expiration (mm)			Thickening Fraction (%)		
	Baseline	Final	*p*-Value	Baseline	Final	*p*-Value
All patients (*n* = 24)	1.1 (0.9–1.2)	1.0 (0.9–1.2)	0.063	0 (0–8.8)	7.2 (0–16.8)	0.209
MV group (*n* = 11)	1.1 (1.0–1.2)	0.9 (0.9–1.1)	0.044	0 (0–0)	0 (0–18.3)	0.042
non-MV group (*n* = 13)	1.0 (0.9–1.2)	1.1 (0.9–1.3)	0.587	8.3 (0–11.1)	7.7 (0–15.0)	0.953

Values are expressed as median (interquartile range). MV, mechanical ventilation.

**Table 3:** Incidence and overlap of diaphragm dysfunction and ICU-AW in each group at final measurements.

	Diaphragm Dysfunction	ICU-AW	Overlap
MV group (*n* = 11)	8 (72.7)	6 (54.5)	6 (54.5)
non-MV group (*n* = 13)	10 (76.9)	3 (23.1)	3 (23.1)

Values are expressed as *n* (%). ICU-AW, ICU-acquired weakness; MV, mechanical ventilation.

**Table 4:** Association between diaphragm dysfunction, ICU-AW, and clinical outcomes in each group.

	MV Group						Non-MV Group					
	Diaphragm Dysfunction			ICU-AW			Diaphragm Dysfunction			ICU-AW		
	Yes (*n* = 8)	No (*n* = 3)	*p*-Value	Yes (*n* = 6)	No (*n* = 5)	*p*-Value	Yes (*n* = 10)	No (*n* = 3)	*p*-Value	Yes (*n* = 3)	No (*n* = 10)	*p*-Value
Hospital mortality	3 (37.5)	0 (0)	0.100	3 (50.0)	0 (0)	0.182	0 (0)	0 (0)	1.000	0 (0)	0 (0)	1.000
Rehabilitation hospital	3 (37.5)	0 (0)	0.464	3 (50.0)	0 (0)	0.018	2 (20.0)	0 (0)	1.000	0 (0)	2 (20.0)	1.000
ICU LOS	11.0 (8.0–26.0)	5.5 (5.3–5.8)	0.145	22.0 (12.8–29.0)	6.0 (5.0–6.0)	0.004	5.0 (3.5–6.5)	4.5 (4.3–4.8)	0.769	4.0 (3.5–6.5)	5.0 (4.0–6.0)	0.811
Hospital LOS	37.0 (16.0–92.0)	15.0 (13.0–17.0)	0.218	79.0 (44.3–92.8)	14.0 (13.0–16.0)	0.004	21.0 (13.5–42.0)	20.0 (18.5–21.5)	1.000	47.0 (30.0–52.0)	19.0 (14.3–32.0)	0.469
Weaningfailure	1 (12.5)	0 (0)	1.000	1 (16.7)	0 (0)	1.000						

Values are expressed as *n* (%). ICU-AW, ICU-acquired weakness; MV, mechanical ventilation; LOS, length of stay.

## Data Availability

The datasets used and/or analyzed in the current study are available from the corresponding author upon reasonable request.
